# Triclosan Impairs Hippocampal Synaptic Plasticity and Spatial Memory in Male Rats

**DOI:** 10.3389/fnmol.2018.00429

**Published:** 2018-11-26

**Authors:** Alejandra Arias-Cavieres, Jamileth More, José Miguel Vicente, Tatiana Adasme, Jorge Hidalgo, José Luis Valdés, Alexis Humeres, Ismael Valdés-Undurraga, Gina Sánchez, Cecilia Hidalgo, Genaro Barrientos

**Affiliations:** ^1^Biomedical Neuroscience Institute, Universidad de Chile, Santiago, Chile; ^2^Centro Integrativo de Biología y Química Aplicada, Universidad Bernardo O’Higgins, Santiago, Chile; ^3^Physiology and Biophysics Program, ICBM, Faculty of Medicine, Universidad de Chile, Santiago, Chile; ^4^Department of Neuroscience, Faculty of Medicine, Universidad de Chile, Santiago, Chile; ^5^Department of Morphofunction, Faculty of Medicine, Universidad Diego Portales, Santiago, Chile; ^6^Pathophysiology Program, ICBM, Faculty of Medicine, Universidad de Chile, Santiago, Chile; ^7^CEMC, Faculty of Medicine, Universidad de Chile, Santiago, Chile

**Keywords:** hippocampus, structural plasticity, Ca^2+^ signals, synaptic transmission, antimicrobial agents

## Abstract

Triclosan, a widely used industrial and household agent, is present as an antiseptic ingredient in numerous products of everyday use, such as toothpaste, cosmetics, kitchenware, and toys. Previous studies have shown that human brain and animal tissues contain triclosan, which has been found also as a contaminant of water and soil. Triclosan disrupts heart and skeletal muscle Ca^2+^ signaling, damages liver function, alters gut microbiota, causes colonic inflammation, and promotes apoptosis in cultured neocortical neurons and neural stem cells. Information, however, on the possible effects of triclosan on the function of the hippocampus, a key brain region for spatial learning and memory, is lacking. Here, we report that triclosan addition at low concentrations to hippocampal slices from male rats inhibited long-term potentiation but did not affect basal synaptic transmission or paired-pulse facilitation and modified the content or phosphorylation levels of synaptic plasticity-related proteins. Additionally, incubation of primary hippocampal cultures with triclosan prevented both the dendritic spine remodeling induced by brain-derived neurotrophic factor and the emergence of spontaneous oscillatory Ca^2+^ signals. Furthermore, intra-hippocampal injection of triclosan significantly disrupted rat navigation in the Oasis maze spatial memory task, an indication that triclosan impairs hippocampus-dependent spatial memory performance. Based on these combined results, we conclude that triclosan exerts highly damaging effects on hippocampal neuronal function *in vitro* and impairs spatial memory processes *in vivo*.

## Introduction

The production and utilization of chemical agents designed to reduce household and human bacterial load is currently on the rise (Levy, 2001; Aiello and Larson, 2003; Dhillon et al., 2015). Many products for personal care and everyday use contain the antimicrobial agent triclosan [5-chloro-2-(2,4-dichlorophenoxy)phenol], a synthetic broad-spectrum bactericide (Singer et al., 2002). Triclosan (TCS) is a lipophilic molecule extensively used that has an annual production of 1,500 tons worldwide (Dhillon et al., 2015). About 85% of the total triclosan global use stems from its presence in personal care products, compared to 5% in textiles and 10% in plastics and food contact materials. Triclosan was removed in 2010 from the European Union list of additives for use in plastic food contact (European Commission. Directorate General for Health and Consumers, 2010), but it is still present in some soaps, toothpastes, cleaning products, bedding, clothes, fabrics, shoes, carpets, plastics, and medical supplies (Rodricks et al., 2010). In 2016, the United States Food and Drug Administration gave manufacturers of consumer soaps 1 year to remove TCS from their products; yet, this deadline did not apply to other personal products such as toothpaste. Furthermore, TCS remains widely used worldwide in many household and personal care products.

Mounting evidence shows that TCS impairs cardiac and skeletal muscle excitation–contraction coupling, thus disturbing normal Ca^2+^ signaling in these tissues (Cherednichenko et al., 2012; Fritsch et al., 2013). Additionally, an association between TCS, tumor promotion in liver, and oxidative stress has been reported (Yueh et al., 2014). A recent study in mice reported that TCS causes colonic inflammation and colitis, alters gut microbiota, and worsens colitis-associated colon cancer (Yang et al., 2018). Triclosan induces Fas-dependent apoptosis in neocortical neurons *in vitro* (Szychowski et al., 2015) and produces toxic effects in neural stem cells through mechanisms involving increased reactive oxygen species (ROS) production and apoptosis (Park et al., 2016), leading to the proposal that TCS acts as a neurotoxic agent (Ruszkiewicz et al., 2017). In consonance with this idea, incubation of primary neocortical neurons with TCS decreases the expression of *N*-methyl-D-aspartate (NMDA) receptor (NMDAR) subunits and enhances NMDAR-dependent ROS generation and caspase-3-dependent apoptosis (Szychowski et al., 2018).

To our knowledge, however, information is not available regarding the effects of TCS on hippocampal synaptic plasticity and spatial memory processes. The hippocampus, which mediates the formation of long-term memories and is critical for memory consolidation, is a well-established model to study neuronal function (Andersen et al., 2006). Hippocampal synapses exhibit both long-term potentiation (LTP) and long-term depression of synaptic strength; these processes are considered the cellular substrates of memory encoding (Bear and Malenka, 1994; Malenka and Bear, 2004; Whitlock et al., 2006; Citri and Malenka, 2008). In particular, LTP in the CA1 region of the hippocampus may be a primary synaptic mechanism underlying specific types of long-term memory processes (Tsien et al., 1996; Gruart et al., 2006; Whitlock et al., 2006). Additionally, changes in synapse/spine morphology, density, and number have been associated with changes in synaptic efficacy and LTP (Sorra and Harris, 1998; Matsuzaki et al., 2001; Lang et al., 2004).

At present, it is widely accepted that hippocampal LTP requires intracellular Ca^2+^ signals and evokes dendritic spine remodeling (Lang et al., 2004). Calcium signaling is considered central for normal neuronal function (Berridge, 1998) and plays a pivotal role in neuronal plasticity (Lynch, 2004). Hippocampal structural plasticity entails dendritic spine remodeling (Kitanishi et al., 2009), which associates with memory mechanisms (Segal, 2017), and engages intracellular Ca^2+^ signals mediated by ryanodine receptor (RyR) Ca^2+^ release channels present in the endoplasmic reticulum (Adasme et al., 2011; Korkotian and Segal, 2011; Grigoryan et al., 2012; More et al., 2018). Given that TCS disturbs normal Ca^2+^ signaling in heart and skeletal muscle (Cherednichenko et al., 2012), it is likely to disrupt Ca^2+^ signaling pathways required for hippocampal neuronal function.

In this work, we report that TCS significantly perturbs hippocampal synaptic plasticity – including LTP and dendritic spine remodeling – and impairs rat performance in a spatial memory task. We conclude, based on these findings, that TCS exerts highly damaging effects on rodent hippocampal neuronal function. If chronic TCS exposure were to produce these damaging effects in the human brain, the presence of TCS in personal care products should be reconsidered.

## Materials and Methods

### Materials

Minimum essential medium, horse serum, serum-free Neurobasal medium, GIBCOTM B27, Fluo-4-AM, and 2 mM GlutamaxTM were from Thermo Fisher Scientific (Waltham, MA). The pRFP-C-RS vector was from Origene (Rockville, MD), and BDNF was from Chemicon Millipore (Darmstadt, Germany). Triclosan was from Sigma (St. Louis, MO, United States). Enrofloxacin was from Bayer (Pittsburgh, PA, United States) and Ketophen from RhodiaMerieux (Santiago, Chile).

### Animals

Male Sprague Dawley rats (8 to 10-week-old) were obtained from the animal facility of the Faculty of Medicine, Universidad de Chile. Food and water were provided *ad libitum*. Animals were maintained in a temperature-controlled room at a 12 h light–dark cycle (lights on at 7 a.m.). All experiments were performed in the light phase. The experimental protocols used in this work complied with the “Guiding Principles for Research Involving Animals and Human Beings” of the American Physiological Society and were approved by the Bioethics Committee on Animal Research, Faculty of Medicine, Universidad de Chile.

### Primary Hippocampal Cultures

At embryonic day, 18 pregnant rats were sacrificed by decapitation under isoflurane anesthesia, and primary cultures were prepared from the hippocampus dissected from the embryos (Adasme et al., 2011). Cells were plated in minimum essential medium plus 10% horse serum for 40 min and were maintained for 14 days at 37°C under 5% CO_2_/95% O_2_ in serum-free Neurobasal medium supplemented with GIBCOTM B27 and 2 mM GlutamaxTM. Primary hippocampal cultures were used at 14 days *in vitro* (DIV).

### Morphological Analysis of Dendritic Spines

Experiments were performed as described earlier (Adasme et al., 2011). Briefly, primary hippocampal cultures were plated over polylysine-coated 35 mm plates (∼500,000 cells/cover). Cultures were transiently transfected at 13 DIV with the pRFP-C-RS vector to visualize cellular structures through red fluorescence detection; 24 h after transfection (14 DIV), cultures were tested for changes in spine density elicited by incubation for 6 h with BDNF (50 ng/ml), as described (Adasme et al., 2011). To evaluate the effects of triclosan on BDNF-induced spine density changes, cultures were pre-incubated for 1 h with 1 μM triclosan prior to addition of BDNF. As control, we added the vehicle DMSO (final concentration <0.01%). Images of dendrites were acquired by confocal microscope (Carl Zeiss, Axiovert 200, LSM 5 Pascal, Jena, Germany) of living cells maintained during the determination in Tyrode medium, under the following conditions: 63× oil objective, 4× digital zoom, NA 1.4, excitation/emission 543/530–600 nm. For spine density determinations, randomly selected dendrites present in 30–50 μm proximity to the soma were analyzed independently; 2–3 dendrites from each one of eight different cultures were subject to blind analysis in each case. The number of spines was quantified in 36.6 μm × 36.6 μm fields, in which dendrite segments were placed in the diagonal (corresponding to 51.7 μm). Dendritic spine density was analyzed by measuring the number of spines present in a dendritic length of 50 μm. The IMAGE J image program (National Institutes of Health, United States) was used for image deconvolution and to construct z-project images from 9–15 stacks (0.4 μm each).

### Detection of Intracellular Ca^2+^ Signals

After 14 days in culture, neurons were transferred to modified Tyrode solution (in mM: 129 NaCl, 5 KCl, 2 CaCl_2_, 1 MgCl_2_, 30 glucose, 25 Hepes, pH 7.3), preloaded for 30 min at 37°C with 2 μM Fluo-4-AM and washed three times with Tyrode solution to allow complete dye de-esterification. These conditions were chosen to avoid deleterious dye effects (Smith et al., 2018). Fluorescence images of intracellular Ca^2+^ signals in primary hippocampal neurons were acquired at 1 s intervals in an inverted epifluorescence microscope (Carl Zeiss, Axiovert A1, Colibri system, Jena, Germany), utilizing the 63× objective and excitation at 470 nm with a LED module. Images from cell bodies were collected and analyzed. Ca^2+^ signals are expressed as Δ*F*/*F*_0_, where *F* and *F*_0_ correspond to the experimental and the basal fluorescence levels, respectively. All experiments were performed at room temperature (∼24°C). Calcium signals were analyzed as described elsewhere (Uhlen, 2004; Maggio and Vlachos, 2014).

### Single Cell Electrophysiology

We evaluated the effect of triclosan on membrane capacity and membrane resistance of primary hippocampal neurons under the cell-attach configuration, as described (Bournaud et al., 1998).

### Slice Preparation

Male rats (3–4 weeks) under isofluorane anesthesia were euthanized by decapitation and their brains were quickly removed. The hippocampus was dissected in cold dissection buffer containing (in mM: 212.7 sucrose, 5 KCl, 1 MgCl_2_, 2 CaCl_2_, 10 glucose, 1.25 NaH_2_PO_4_, 26 NaHCO_3_, pH 7.4) and was cut into 400 μm transversal slices using a vibratome (Vibratome 1000 plus, Ted Pella Inc., CA, United States), as detailed elsewhere (Munoz et al., 2011). Hippocampal slices were transferred to an immersion storage chamber kept at room temperature in artificial cerebrospinal fluid (ACSF) containing (in mM: 124 NaCl, 5 KCl, 1.25 NaH_2_PO_4_, 1 MgCl_2_, 2 CaCl_2_, 10 glucose, 26 NaHCO_3_, pH 7.4), in 95% O_2_/5% CO_2_. Slices were kept in this solution for at least 1 h before recording at 30 ± 2°C. Stock triclosan solutions (100 mM) were prepared in dimethylsulfoxide (DMSO). Hippocampal slices were incubated with ACSF solutions containing 1, 5, or 10 μM triclosan or vehicle (up to 0.01%, DMSO) as control.

### Hippocampal Electrophysiology

Electrophysiological experiments were performed in an immersion-recording chamber. To evaluate field excitatory postsynaptic potentials (fEPSPs), hippocampal slices were superfused with ACSF (in 95% O_2_/5% CO_2_) at a rate of 2 ml/min at 30 ± 2°C. fEPSP, evoked by square current pulses (0.2 ms) delivered with a concentric bipolar stimulating electrode (FHC Inc., Bowdoinham, ME, United States) located in the Schaeffer collateral–commissural fibers, were recorded using glass microelectrodes (2–3 MΩ) filled with ACSF placed into the *stratum radiatum* of the CA1 region. To evaluate basal excitatory synaptic transmission, pulses of 25, 50, 75, 100, 150, and 200 μA were applied to construct an input/output curve. Results are presented as stimulus intensity versus fiber volley (FV) amplitude, or as stimulus intensity versus fEPSP slope. To evaluate pre-synaptic components of the responses, we used the following paired-pulse stimulation protocol: two pulses were applied every 15 s, with inter-stimulus intervals starting with 20 ms and ending with 640 ms, doubling the interval after each trial. The results are presented as the ratio between the initial fEPSP slopes evoked by the second over the first stimulus. After monitoring both basal synaptic transmission and pre-synaptic responses, we evaluated LTP adjusting the stimulus intensity to generate fEPSPs to half of the maximal evoked response, with pulses applied every 15 s until a stable baseline was attained for at least 15 min. To induce LTP, we applied the TBS protocol, consisting of 4 trains of 10 bursts at 5 Hz each (1 burst = 4 pulses at 100 Hz). In all experiments, fEPSP recordings were continued for 60 min after applying the TBS protocol. Recordings were filtered at 10 kHz and were digitized at 5 kHz, using Igor Pro (WaveMetrics Inc., Lake Oswego, OR, United States). Synaptic responses, quantified as the initial slope of the evoked fEPSPs, were plotted as percentage of basal change relative to the slope of the baseline record, considered as 100%. Triclosan stocks (100 mM) were prepared fresh in dry DMSO and were diluted in aqueous ACSF solution to the indicated concentration.

### Cannulation Surgery

Male Sprague-Dawley rats (2.5 months average age) weighing 230–250 g were used in these experiments. Animals were maintained with a light/dark cycle of 12 h, at an average temperature of 22°C with food and water *ad libitum*, and were handled daily for 2 weeks prior to surgery. Rats were anesthetized prior to surgery with isoflurane in oxygen (2.5% for induction, 1.5% for maintenance; 1 L/min oxygen flow). Sedation depth was monitored by the absence of the toe pinch withdrawal reflex. Cannulas were placed in all rats used in this work (control and TCS-injected). The animal head was restrained with a stereotaxic frame; an incision on the skin and a small craniotomy was conducted to implant two bilateral stainless-steel cannula guides (21-gauge, plastics one). To target the dorsal CA3 region of the hippocampus, we used the following stereotaxic coordinates according to the rat brain atlas (Paxinos and Watson, 2007): anteroposterior: 2.5 mm; laterality ±3.5 mm and 2.7 mm in depth. Cannulas were fixed to the skull using as anchors jewelry stainless steel screws set with dental acrylic. Antibiotic (Enrofloxacin 5%, 19 mg/kg i.p.) and anti-inflammatory (Ketophen 0.2 mg/kg i.p.) drugs were administered at the end of surgery and during three consecutive days.

### Oasis Maze Task

To evaluate hippocampal-dependent spatial memory, we used the Oasis maze task, a modified dry-land version of the Morris water maze (Kesner et al., 1991; Clark et al., 2005; Martinez et al., 2016; More et al., 2018). In brief, the task consisted of a circular open field arena of 1.4 m in diameter containing 21 evenly spaced wells (4.5 cm diameter, 2 cm height), placed 50 cm above the floor, and limited by a wall of 20 cm in height. All experiments were conducted in a dedicated room with distal visual cues. Rats were first exposed for three consecutive days to pre-training sessions, during which rats – water-deprived for 23 h – were trained to seek water-containing wells. To this aim, the animals were allowed to explore the arena for 10 min, in conditions in which all wells contained one drop of water (∼50 μL). The following day, the animals were surgically implanted with injection cannulas placed in the CA3 hippocampal region (Supplementary Figure S2, left panel). After a recuperation period of 7 days, the pre-training sessions were repeated for two consecutive days. The following day, rats were tested in the spatial memory task, which entailed one daily session over six consecutive days; each session comprised 15 trials of up to 1 min duration. Before each session, rats were enclosed within a black cylinder (22 cm diameter, 27 cm height). The trial started after placing the rat in the arena and removing the cylinder, and it ended when the rat reached the baited well or at the end of the 1-min exploration time. An inter-trial interval of 20–30 s was used. During this inter-trial period, the animal was enclosed with the cylinder and was gently moved to a different starting position randomly assigned to prevent stereotyped trajectory or procedural learning of the animal to solve the maze. The reward was maintained every day in the same location.

All animals were tested for three consecutive days. Two hours after completing the training session on the third day, one group of animals (*n* = 6) was injected with TCS (0.5 μl of 10 μM, equivalent to 5 pmol) or with 0.5 μl of saline (Supplementary Figure S2, left panel). Triclosan or saline injection was repeated later on the same day and on the following day. Considering that the rat hippocampus has a volume of ∼0.8 ml (Le Duigou et al., 2005) and assuming homogenous TCS distribution into this hippocampal volume, each TCS injection would yield a final TCS concentration of ∼6 nM. Rats were tested in three consecutive daily sessions following TCS injections. Animal behavior was recorded with a video camera placed in the zenithal position. Off-line analysis of the videos was conducted by using a custom-made MATLAB routine to reconstruct the trajectory of the animal (More et al., 2018). Three behavioral parameters of learning were quantified: hit ratio (number of correct hits in each 15 trial session); latency, defined as the time taken to find the baited well, and distance ratio, defined as the ratio between the observed and the straight path lengths from the starting point to the reward.

### Western Blot Analysis

Five hippocampal slices (400 μm each) from each animal were combined, and extracts were prepared as described (Arias-Cavieres et al., 2017). Proteins were resolved by SDS-PAGE using 3.5–8% Tris-acetate gels for RyR2 determination or 10% gels for assaying Synapsin I and CaMKII protein content and phosphorylation status. Following SDS-PAGE, proteins were transferred to polyvinylidenedifluoride (PVDF) membranes and were probed with one of the following antibodies: anti-RyR2 mouse monoclonal antibody (1:1,000; Thermo Scientific, Rockford, IL, United States), anti-CamKII (pan) (D11A10) rabbit monoclonal antibody (#4436, 1:3,000; Cell Signaling Technology, Danvers, MA, United States). PhosphoCaMKII (phospho T286) rabbit polyclonal antibody (ab32678, 1:5,000), anti-Synapsin I rabbit polyclonal antibody (ab64581, 1:2,500), and anti-Synapsin I (phospho-S603) rabbit polyclonal antibody (ab13879, 1:1,500) were from Abcam (Cambridge, MA, United States). Image acquisition and densitometric analysis of band density were performed, respectively, by means of the Chemidoc^TM^ MP System (Bio-Rad laboratories, Hercules, CA, United States), and the Image Lab software.

### Statistical Analysis

The type of analysis used in each experimental determination is described in detail in each figure legend. Statistical significance was considered at *p* < 0.05. The statistical Power was calculated with alpha = 0.05, using the Sigma Plot program.

## Results

### Effects of Triclosan on Basal Synaptic Transmission

**FIGURE 1:**
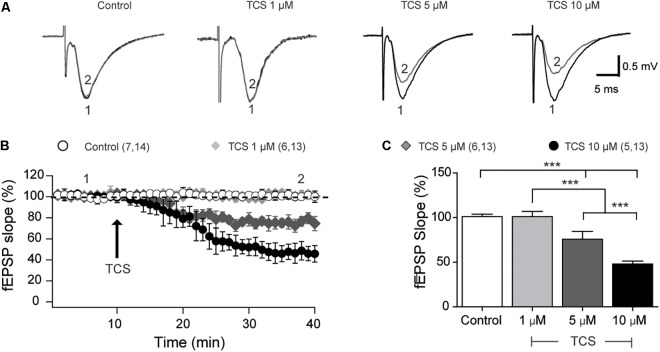
Concentration-dependent effects of triclosan on basal synaptic transmission (CA3-CA1). **(A)** Representative fEPSP traces recorded in hippocampal slices treated with different TCS concentrations, as indicated in the figure. The numbers indicated in the traces correspond to the times of record collection, as indicated in **(B)**. **(B)** Time course records showing the effects of TCS addition (arrow) on basal transmission; numbers in parenthesis represent the number of animals followed by the number of slices used in each determination. **(C)** Bar graph showing average values of fEPSP slopes, collected at the end of the respective records. All values represent Mean ± SE; control (*n* = 7), 1 μM TCS (*n* = 6), 5 μm TCS (*n* = 6), and 10 μm TCS (*n* = 5). Statistical analysis was performed by two-way ANOVA followed by Bonferroni’s *post hoc* test; ^∗∗∗^*p* < 0.001; statistical power: 1.0.

We evaluated the effects of TCS (1, 5, and 10 μM) on hippocampal basal transmission in the CA3-CA1 hippocampal circuit. As illustrated by the representative records of field excitatory postsynaptic potentials (fEPSP) shown in Figure 1A, addition of 1 μM TCS to acute hippocampal slices did not modify fEPSP slopes and basal synaptic transmission (Figure 1B). In contrast, addition of 5 or 10 μM TCS significantly decreased both the slope of the fEPSP records (Figure 1A) and the basal response (Figure 1B). The average values of fEPSP slopes – collected 30 min after TCS addition – are summarized in Figure 1C. Based on these results, we conclude that TCS concentrations ≥5 μM exert significant inhibitory effects on basal synaptic transmission.

### Effects of Triclosan on Fiber Volley Amplitude, Input/Out Responses, and Paired-Pulse Facilitation

To further analyze whether 1 μM TCS impairs other synaptic properties, we measured fiber volley amplitude (FV), a parameter that reflects the number of fibers activated as a function of stimulus intensity (Bodhinathan et al., 2010; Ardiles et al., 2012). Representative traces recorded in control slices or in slices incubated with 1 μM TCS for 15 min are presented in Supplementary Figure S1A. Quantification of FV amplitude versus stimulus intensity showed that slices treated with 1 μM TCS displayed FV amplitude values undistinguishable from controls (Supplementary Figure S1B), an indication that TCS does not interfere with pre-synaptic fiber recruitment. Next, we evaluated the effects of TCS on fEPSP slopes determined at increasing stimulus intensities and assessed whether TCS affected paired-pulse facilitation responses (Schulz et al., 1994). When stimulated at <150 μA slices treated with 1 μM TCS displayed similar fEPSP slopes as controls, but exhibited reduced responses when stimulated at 150 or 200 μA (Supplementary Figure S1C). Slices treated with 1 μM TCS for 15 min displayed paired-pulse facilitation responses undistinguishable from controls (Supplementary Figure S1D), an indication of unaffected presynaptic transmitter release.

### Triclosan Impairs the LTP Response

**FIGURE 2:**
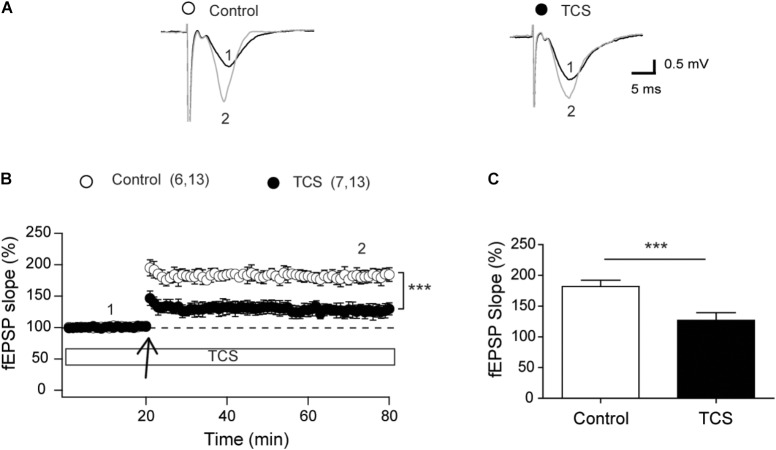
Triclosan impairs LTP (CA3-CA1). **(A)** Representative fEPSP traces recorded in control or slices treated with 1 μM TCS (added 5 min after starting the record). Representative fEPSP traces were collected 10 min before applying the TBS protocol (number 1 in graph B) and 60 min after TBS (number 2 in graph B). **(B)** The LTP-inducing TBS protocol (four trains) was delivered at the time indicated by the arrows. Open circles: Control; black circles: 1 μM TCS. Values presented in each trace represent Mean ± SE; control (*n* = 6); TCS-treated (*n* = 7). **(C)** Average values of the fEPSP slopes recorded during the last 10 min of the records; open bar: control; solid bar: treated with 1 μM TCS. Statistical data analysis was performed with the Mann–Whitney *U*-test; ^∗∗∗^*p* < 0.001.

To characterize the effects of TCS on neuronal synaptic plasticity, we tested the effects of 1 μM TCS, a concentration that did not affect fEPSP slopes, basal synaptic transmission, fiber volley amplitude, or paired-pulse facilitation responses, on LTP induced by the theta-burst stimulation (TBS) protocol (Larson et al., 1986; Raymond, 2007). Representative fEPSP traces recorded before (1) and ∼60 min after applying the TBS protocol (2) show significant reduction in the responses recorded at 60 min in TCS-treated slices (Figure 2A). Addition of 1 μM TCS to slices 15 min before TBS caused significant reduction in the LTP response relative to controls (Figure 2B). Average values of the responses collected 60 min after LTP induction (Figure 2C) showed that slices treated with 1 μM TCS displayed (in %) significantly lower values (122.6 ± 10.6) relative to controls (182.0 ± 10.2). These results show that 1 μM TCS impairs LTP presumably by interfering with postsynaptic pathways.

### Triclosan Does Not Affect the Passive Electrical Membrane Properties of Neuronal Cells

Triclosan is a highly hydrophobic molecule (DeSalva et al., 1989) likely to partition into the lipid component of cellular membranes. We found that incubation of primary hippocampal cultures for 20 min with 5 μM TCS, a concentration of TCS that significantly decreased both the slope of the fEPSP records and basal synaptic transmission (Figure 1), did not have significant effects on neuronal membrane capacity (pF, C: 35.8 ± 8.4, *n* = 12; TCS: 26.0 ± 4.4) or membrane resistance (MΩ, C: 98.8 ± 13.9, *n* = 12; TCS: 103.0 ± 13.2, *n* = 11).

### Triclosan Inhibits Both Dendritic Spine Remodeling and Spontaneous Ca^2+^ Oscillations in Primary Hippocampal Cultures

**FIGURE 3:**
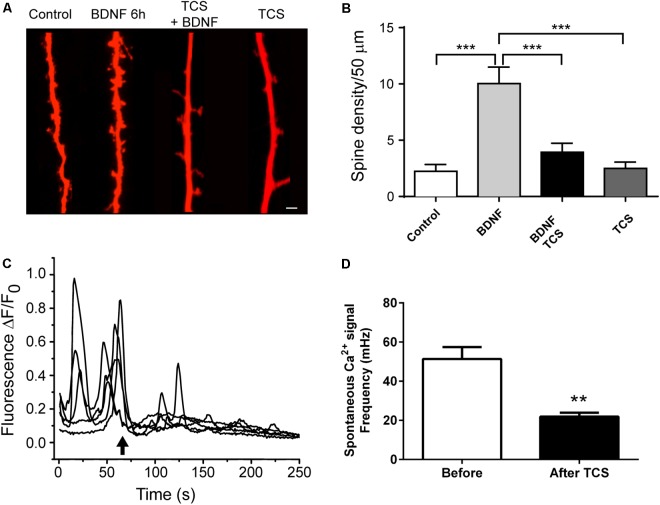
Triclosan prevents BDNF-induced spine remodeling in hippocampal neurons and disrupts spontaneous Ca^2+^ oscillations. **(A)** Representative images of neuronal projections acquired from control cultures (plus or minus TCS) or from cultures treated with BDNF (plus or minus TCS). Cultures were pre-incubated with TCS (1 μM) for 30 min before subsequent incubation with BDNF (50 ng/mL) or vehicle for 6 h. Scale bar, 5 μm. **(B)** Quantification of spine density (Mean ± SE, *n* = 8). Statistical analysis was performed with One-way ANOVA; ^∗∗∗^*p* < 0.001; Power = 0.99. **(C)** Representative recordings of the spontaneous Ca^2+^ oscillations in neurons loaded with the Ca^2+^ probe Fluo-4. Triclosan (1 μM) was added at the arrow. **(D)** Quantification of the frequency of oscillations measured before and after TCS addition (Mean ± SE, *n* = 5). The statistical analysis of spine remodeling data was performed by one-way ANOVA followed by Bonferroni’s *post hoc* test. Calcium oscillation data were compared using Student’s paired *t*-test. ^∗∗^*p* < 0.01; statistical power = 0.84.

Synaptic plasticity encompasses structural plasticity; a process characterized by dendritic spine remodeling that entails generation and growth of dendritic spines (Kitanishi et al., 2009). We tested whether incubation with 1 μM TCS, which impaired the LTP response, also affected structural plasticity. To this aim, we determined if incubation with 1 μM TCS affected the spine remodeling induced by incubation of primary hippocampal cultures for 6 h with brain-derived neurotrophic factor (BDNF), a neurotrophin known to promote dendritic spine remodeling (Tyler and Pozzo-Miller, 2001). As illustrated by the representative images (Figure 3A) and the average results from four independent experiments (Figure 3B), pre-incubation for 30 min with 1 μM TCS, which was maintained during the subsequent 6-h incubation period with BDNF or vehicle, abolished BDNF-induced spine remodeling but did not modify basal spine density.

Previous work showed that TCS disrupts voltage-dependent Ca^2+^ signals in skeletal and cardiac muscle (Cherednichenko et al., 2012). Addition of 1 μM TCS (arrow) to primary hippocampal cultures (Figure 3C) caused a significant inhibition of the spontaneous Ca^2+^ oscillations displayed by hippocampal neurons before TCS addition (Figure 3D). The implications of these results are addressed in the Discussion section.

### Triclosan Impairs Hippocampus-Dependent Spatial Navigation and Memory

**FIGURE 4:**
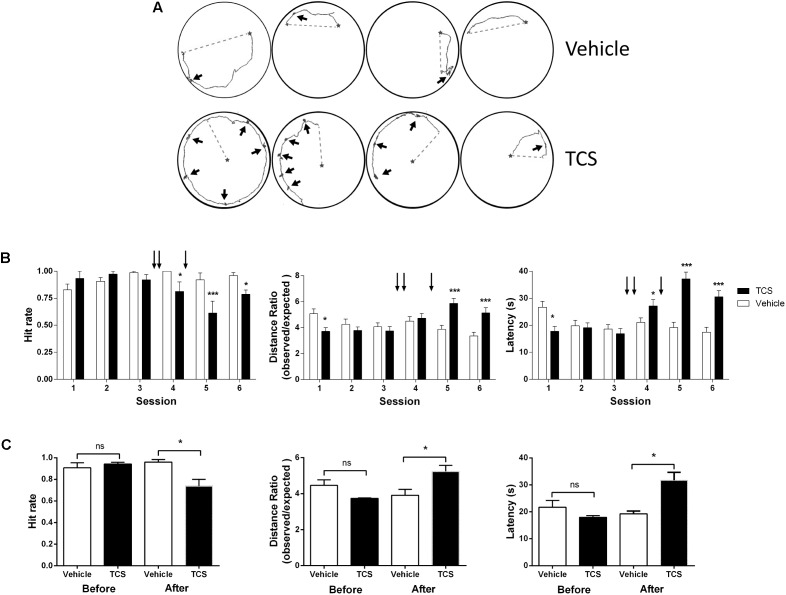
Triclosan impairs hippocampus-dependent spatial memory. Triclosan (0.5 μl, 10 μM) was injected three times into the CA3 region of rat hippocampus (Supplementary Figure S2, left panel), after which animals were trained in a spatial memory task (for details, see text). **(A)** Representative experiment showing how the animals explored the maze during the Oasis maze task. The animal location was tracked by video recordings and was analyzed using a MATLAB routine. Walked (solid lines) and expected/shortest (dotted lines) distance records were acquired in session 5 (see Supplementary Figure S2, left panel). The black stars indicate the bait location. The thicker regions of the tracking solid line correspond to periods where the animal remained rotating in the same place (black arrows). **(B)** The arrows over each graph indicate the times of TCS injections. Left panel: Hit rate, defined as the relative ratio between the successful hits and the 15 trials performed in each of six daily sessions. Center panel: Latency, defined as the time it takes the animal to find the reward. Right panel: Ratio between the distance covered by the animal and the shortest linear distance to the baited well. **(C)** Average values of hit rates, latencies, and distance ratios evaluated in sessions 1–3, and sessions 4–6 in TCS-injected or vehicle-injected rats. Statistical analysis in **(B)** was performed using two-way ANOVA followed by Holm-Sidak *post hoc* test. Statistical analysis in **(C)** was performed using paired Student’s *t*-test. Values represent Mean ± SE (*n* = 6); ^∗^*p* < 0.05; ^∗∗∗^*p* < 0.001; statistical power = 1.0.

The LTP response is currently considered a substrate of memory processes (Morris et al., 1986). The LTP inhibition produced by TCS led us to test whether TCS affects spatial memory formation in rats. To this aim, we injected TCS into the CA3 region of rat hippocampus (Supplementary Figure S2) and evaluated spatial performance in the Oasis maze task (see section “Materials and Methods”). The representative experiment shown in Figure 4A illustrates the remarkable effects of TCS on rat performance in the Oasis maze task. The TCS-injected rat displayed noticeably longer navigation trajectories than the control rat in three separate trials recorded at 5, 10, and 15 min during session 4 and exhibited many periods in which the animals stayed in the same place undergoing rotating movements (black arrows); control animals rarely displayed this behavior along their trajectories. In addition, TCS injections significantly impaired hit rates, latency times, and the distance traveled to the reward relative to the controls (Figures 4B,C), typical parameters used to assess navigation in the Oasis maze task (Clark et al., 2005; More et al., 2018). Rats injected with TCS displayed similar exploration velocities as vehicle injected rats (Supplementary Figure S2, right panel), an indication that TCS did not affect rat motor ability. Of note, TCS injection deteriorated the spatial performance of rats in the Oasis maze despite the fact that before injection the same rats successfully navigated and effectively learned to find the baited well (Figure 4C).

Based on these combined results, we strongly suggest that TCS exerts severe negative effects on activity-dependent hippocampal neuronal function and on spatial memory performance.

### Triclosan Promotes RyR2 Downregulation

**FIGURE 5:**
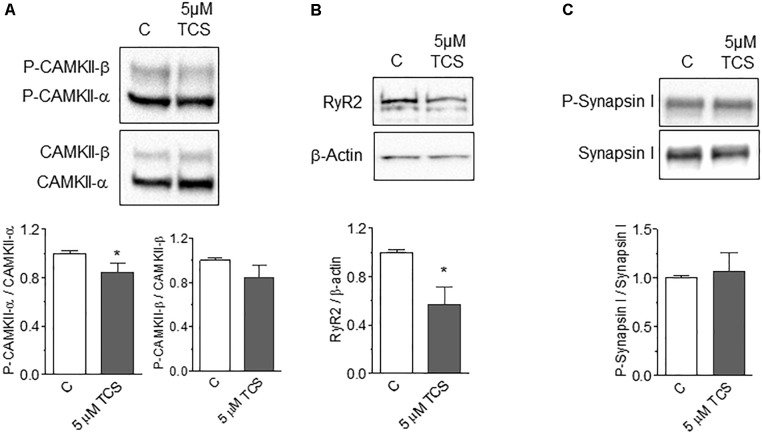
Phosphorylation levels of CaMKII and Synapsin I and RyR2 protein content in control and TCS-injected rat hippocampus. **(A)** Representative Western blots and quantification of phosphorylation levels of CaMKII-α and CaMKII-β assessed in control and TCS-incubated (30 min, 5 mM) hippocampal slices. **(B)** Representative Western blots and quantification of RyR2 protein levels in control and TCS-incubated (30 min, 5 mM) hippocampal slices. **(C)** Representative Western blots and quantification of phosphorylation levels of Synapsin I assessed in control and TCS-incubated (30 min, 5 μM) hippocampal slices. Statistical analysis was performed with unpaired Student’s *t*-test. Values represent Mean ± SE (*n* = 4); ^∗^*p* < 0.05. CaMKII: statistical power = 0.35; RyR2: statistical power = 0.68.

A previous study reported that prolonged incubation (≥3 h) of primary neocortical neurons with 10 μM TCS reduced the protein expression of several NMDAR sub-units (Szychowski et al., 2018). Hence, we tested whether TCS modified the protein contents of two NMDAR downstream targets, the CaMKII enzyme (Bayer et al., 2001) and RyR channels (Riquelme et al., 2011). To this aim, we used 5 μM TCS, a concentration that caused ∼20% inhibition of fEPSP slopes (Figure 1). Acute hippocampal slices incubated for 30 min with 5 μM TCS did not display changes in the protein content of CaMKII-α or CaMKII-β, but caused a modest but significant reduction of CaMKII-α phosphorylation levels while CaMKII-β phosphorylation levels did not change (Figure 5A). In addition, incubation with TCS caused a significant reduction of RyR2 protein content (Figure 5B), the predominant RyR isoform expressed in rat hippocampus that has a central role in synaptic plasticity and memory processes (More et al., 2018). In contrast, the protein content and the phosphorylation levels of the presynaptic protein Synapsin I did not change following incubation of acute hippocampal slices with 5 μM TCS for 30 min (Figure 5C).

## Discussion

### Summary of Results

In this work, we report that low concentrations of TCS (1–5 μM) affected hippocampal neuronal function at various levels. In particular, TCS disrupted hippocampal synaptic plasticity, evidenced by impaired TBS-prompted LTP induction and defective BDNF-induced dendritic spine remodeling, and also reduced CaMKII-α phosphorylation levels and RyR2 protein content in acute hippocampal slices. In addition, primary hippocampal neurons exposed to TCS displayed a significant decrease in the frequency of spontaneous Ca^2+^ signals. Moreover, TCS injected intra-hippocampus (CA3) caused marked defects in spatial memory performance. Altogether, these results provide novel information on the deleterious effects of TCS on rodent neuronal Ca^2+^ signaling, synaptic plasticity, and spatial memory processes.

### TCS Impairs Hippocampal LTP, Structural Plasticity, and Spatial Memory

Synaptic plasticity denotes changes in the efficacy of synaptic transmission in response to neuronal activity (Bliss et al., 2014); it also promotes related structural plasticity responses exemplified by dendritic spine remodeling (Bailey et al., 2004). Here, we report that acute exposure to a low TCS concentration (1 μM) markedly inhibited LTP induction in hippocampal slices and significantly decreased BDNF-induced dendritic spine remodeling in primary hippocampal neurons. Rodent models of depression present a reduction in dendrite complexity and spine density in the hippocampus (Duman and Aghajanian, 2012). Accordingly, future studies should address if chronic TCS exposure impairs activity-dependent dendritic spine remodeling in human hippocampal neurons, since this reduction might be a contributing factor to human depression.

Present evidence supports LTP and structural plasticity as the biological substrates for associative learning and long-term memory (Bliss and Collingridge, 1993; Martin et al., 2000; Leuner et al., 2003; Bailey et al., 2004, 2013; Lynch, 2004; Gruart et al., 2006; Whitlock et al., 2006; Fedulov et al., 2007; Holtmaat and Svoboda, 2009; Kasai et al., 2010; Giachero et al., 2013; Baudry et al., 2015; Lynch et al., 2015). In agreement with the LTP and structural plasticity impairments caused by TCS, we found that after three intra-hippocampal TCS injections, which presumably yielded a final hippocampal TCS concentration of up to ∼18 nM (see Materials and Methods section), rats displayed significant defects in the performance of a spatial memory task.

### TCS Impairs Hippocampal Ca^2+^ Signaling

Here, we report that exposure of primary hippocampal neurons to TCS caused a significant decrease in the frequency of spontaneous Ca^2+^ signals. Addition of up to 10 μM TCS to RyR channels from brain cortex incorporated in planar lipid bilayers does not affect RyR single channel properties (Bull et al., manuscript in preparation). Based on these combined results, we suggest that TCS disturbs Ca^2+^ entry pathways required for neuronal calcium-dependent responses, including CamKII-α phosphorylation, LTP, dendritic spine remodeling, and hippocampus-dependent spatial memory. Studies showing that TCS disrupts Ca^2+^ signaling in both cardiac and skeletal muscle by inhibiting Ca^2+^ currents mediated by voltage-dependent Ca^2+^ channels (Cherednichenko et al., 2012), support this proposal. Of note, TCS did not modify two pre-synaptic responses, paired-pulse facilitation, and Synapsin I phosphorylation. Therefore, we propose that TCS primarily affects postsynaptic Ca^2+^ signaling pathways.

These novel findings complement and expand a recent report showing that neocortical neurons in primary culture exhibit decreased protein levels of several NMDAR subunits after 3, 6, or 24 h post-treatment with 10 μM TCS (Szychowski et al., 2018). In particular, the significant RyR2 protein decrease induced by TCS reported here, which occurred within minutes after TCS addition, may contribute to the defective spatial memory displayed by TCS-injected rats since a decrease in RyR2 expression markedly impairs this process (More et al., 2018). Therefore, we propose that TCS disrupts Ca^2+^ signaling pathways required for synaptic plasticity and memory processes (Berridge et al., 2003; Maggio and Vlachos, 2014).

Disruption of neuronal Ca^2+^ signaling has deleterious effects on experience-dependent dendritic plasticity during rat development (Lohmann and Wong, 2005; Yang et al., 2009; More et al., 2018) and perturbs synaptic plasticity responses (Zundorf and Reiser, 2011; Pchitskaya et al., 2017). There is evidence linking anomalous intracellular Ca^2+^ signaling with autism spectrum disorder (Gargus, 2009; Vallipuram et al., 2010; Wayman et al., 2012; Shen et al., 2015), Alzheimer’s disease (Mattson and Chan, 2001; Berridge et al., 2003; Paula-Lima et al., 2011; Popugaeva et al., 2018), and other neurodegenerative diseases (Mattson and Chan, 2001; Bezprozvanny, 2010). Through disruption of normal neuronal Ca^2+^ signaling, chronic TCS exposure may contribute to cause the memory defects associated with these pathological conditions.

### Presence of TCS in Human and Animal Tissues

Several reports indicate that TCS accumulates in experimental animals (Pannu et al., 2012), as well as in liver and kidney samples from birds (Tanoue et al., 2014), and distributes into the whole body, including the brain of experimental animals exposed to commercial forms of TCS (Fang et al., 2016). Earlier works reporting that TCS did not accumulate in human tissues (Bagley and Lin, 2000; Lin, 2000), suggested that TCS is metabolized and eliminated from the body. Subsequent studies refuted these earlier reports by showing the presence of TCS in human adipose tissue (Wang et al., 2015), breast milk (Allmyr et al., 2006), and brain tissue (Geens et al., 2012). Moreover, a study performed on 181 pregnant women showed that TCS was present in all urine samples, as well as in 51% of cord blood samples (Pycke et al., 2014); these findings raise the possibility that intrauterine TCS exposure may affect brain development and function at early embryonic stages. Likewise, a recent United States study carried out in 12,793 individuals reported that ∼98% of them had TCS concentrations in the 30 nM – 2 μM range (mean = 0.6 μM) in their urine (Pycke et al., 2014). Other studies reported that in the Chinese population TCS was present in 80% of 209 tested subjects (Yin et al., 2016), while in Australia TCS was detected in all urine samples of 2,400 tested subjects (Heffernan et al., 2015). These results show that TCS presence in human urine is a worldwide and common occurrence, which reflects significant exposure of the human population to TCS. The highly lipophilic properties of TCS render it readily absorbable by mucous membranes (Lin, 2000), skin (Moss et al., 2000; Chedgzoy et al., 2002), and following oral intake (Bagley and Lin, 2000; Lin, 2000; Sandborgh-Englund et al., 2006; Weatherly and Gosse, 2017). After oral administration, TCS plasma levels increase rapidly reaching the maximum concentration in ∼1–3 h, with a terminal plasma half-life of ∼21 h; the accumulated urinary excretion ranges from 24 to 83% of the oral dose within the first 4 days after TCS administration (Sandborgh-Englund et al., 2006). In humans, TCS percutaneous absorption is estimated to be around 6% of the exposure dose, and a main proportion of the absorbed TCS is excreted in the urine within the first day after the exposure (Queckenberg et al., 2010).

Triclosan is present in several commercial products at a concentration of ∼15 mM. Therefore, nanomolar TCS concentrations may be present in the brain after daily and multiple exposures to products that contain this chemical, such as toothpastes and soaps. Consequently, and considering the widespread exposure to TCS-containing personal care products – plus the fact that this chemical has been found in dust from private houses and workplaces (Canosa et al., 2007; Geens et al., 2009) – we posit that the presence of TCS in household products may have deleterious effects on human neurological health. Nevertheless, although TCS has been found with low frequency in human post-mortem brain tissue (Geens et al., 2012), to our knowledge direct determinations of TCS concentrations in human cerebrospinal fluid (CSF) have not been reported. Consequently, it is not possible to discern how TCS concentrations detected in human urine samples correlate with TCS brain levels. We propose that in order to ascertain whether TCS levels in CSF correlate with clinical neurological performance and neurological diseases, the actual TCS concentrations in human CSF samples should be evaluated. These determinations would provide sound information to evaluate the safety of TCS presence in daily use products.

## Author Contributions

AA-C performed the electrophysiology experiments in slices, analyzed the data, and contributed to manuscript writing. JM and JV performed the behavioral experiments and analyzed the data. TA determined the dendritic spine changes and analyzed the data. JH performed the single-cell electrophysiological experiments and analyzed the data. JV performed the behavioral experiments and analyzed the data. AH contributed to manuscript writing. IV-U performed the electrophysiology experiments in slices and analyzed the data. GS performed the Western blot experiments and analyzed the data. CH designed the experiments, analyzed the results, and wrote the final version of the manuscript. GB designed the experiments, performed the neuronal Ca^2+^ determinations and electrophysiology experiments in slices, analyzed the data, and wrote the final version of the manuscript.

## Conflict of Interest Statement

The authors declare that the research was conducted in the absence of any commercial or financial relationships that could be construed as a potential conflict of interest.
