# Supplementary material for: Triclosan Impairs Hippocampal Synaptic Plasticity and Spatial Memory in Male Rats
Source: Front Mol Neurosci. 2018 Nov 26;11:429. doi: 10.3389/fnmol.2018.00429 (PMC6275195; doi:10.3389/fnmol.2018.00429)
Supplement: Supplementary file 1 [file Table_1.DOCX]

**SUPPLEMENTARY MATERIAL**


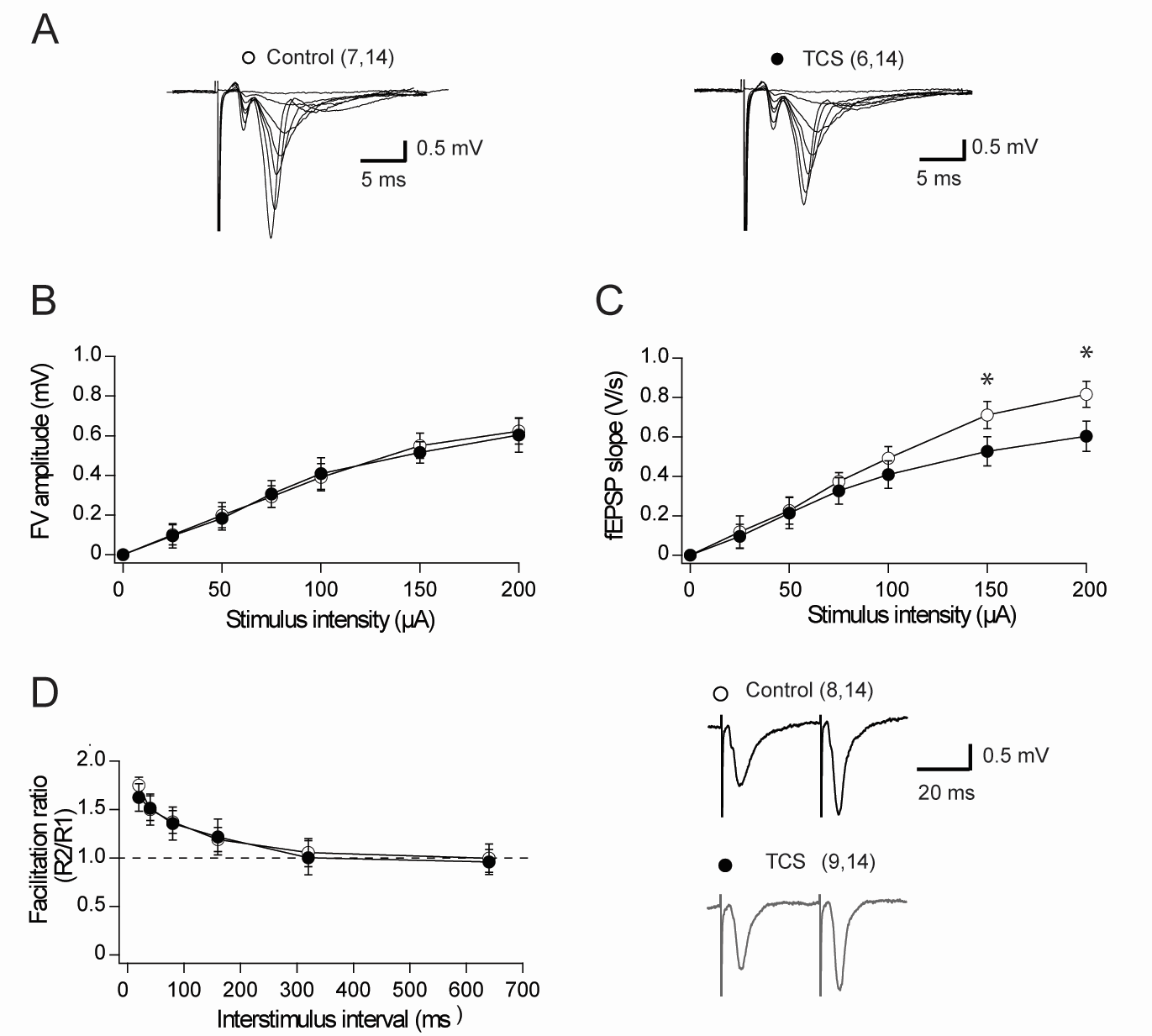


**S1. (Supplementary Figure 1). Effects of triclosan on fiber volley amplitudes, input-output responses and** **paired pulse facilitation. A:** Representative fEPSP traces recorded in control or slices pre-incubated for 15 min with 1 µM triclosan. **B**: Fiber volley amplitude as a function of stimulus intensities recorded in control or slices pre-incubated for 15 min with 1 µM triclosan. **C.** Input-output responses, showing fEPSP slope values elicited by increasing stimulus intensities recorded in control or slices pre-incubated for 15 min with 1 µM triclosan. **D**. The left panel shows the paired-pulse facilitation responses, recorded in control or slices pre-incubated for 15 min with 1 µM triclosan. Open circles: control; black circles: 1 µM triclosan. The right panel shows representative traces recorded at inter-stimulus intervals of 40 ms. Statistical analysis was performed using Mann–Whitney U test. Values represent Mean ± SE (n = 4); *: p < 0.05.

**

**

**S2. (Supplementary Table and Figure 2). Schedule of injections and navigation velocities recorded in control and TCS-injected rats. Left panel:** Table showing the schedule of training sessions and TCS injections. **Right panel:** Navigation velocities recorded in rats before (sessions 1-3) or after (session 5 and 6) vehicle- or after completion of TCS injections. Data were analyzed using One-way ANOVA. Values represent Mean ± SE (n = 6). Data were analyzed using One-way ANOVA; no significant differences between values were found.
